# Prediction of Inpatient Rehabilitation Length, Discharge Destination and Home-Care Needs After Total Hip and Knee Arthroplasty for Osteoarthritis: A Follow-Up Study on 1.679 Patients

**DOI:** 10.3390/jcm15062294

**Published:** 2026-03-17

**Authors:** Federico Pennestrì, Giuseppe Banfi, Catia Pelosi, Dario Grippa, Marta Valenti, Lucia Imperiali, Stefano Borghi, Stefano Negrini, Carlotte Kiekens, Valentina Tosto, Claudio Cordani, the PREPARE Project Group

**Affiliations:** 1 Scientific Direction, IRCCS Galeazzi-Sant’Ambrogio Hospital, Via Belgioioso 157, 20157 Milan, Italy; banfi.giuseppe@hsr.it (G.B.); valentina.tosto94@gmail.com (V.T.); 2 Faculty of Medicine and Surgery, Vita-Salute San Raffaele University, Via Olgettina 58, 20132 Milan, Italy; 3 Specialistic Rehabilitation Unit, IRCCS Galeazzi-Sant’Ambrogio Hospital, Via Belgioioso 157, 20157 Milan, Italy; medicalcentersnc@gmail.com (C.P.); dario.grippa@grupposandonato.it (D.G.); 4 Information Technology Unit, IRCCS Galeazzi-Sant’Ambrogio Hospital, Via Belgioioso 157, 20157 Milan, Italy; marta.valenti@grupposandonato.it; 5 Laboratory of Movement and Sport Science, IRCCS Galeazzi-Sant’Ambrogio Hospital, Via Belgioioso 157, 20157 Milan, Italy; lucia.imperiali@grupposandonato.it (L.I.); stefano.borghi@grupposandonato.it (S.B.); 6 Evidence-Based Rehabilitation Lab., IRCCS Galeazzi-Sant’Ambrogio Hospital, Via Belgioioso 157, 20157 Milan, Italy; stefano.negrini@unimi.it (S.N.); carlotte.kiekens@isico.it (C.K.); claudio.cordani@grupposandonato.it (C.C.); 7 Department of Biomedical, Surgical and Dental Sciences, University of Milan “La Statale”, Via Festa del Perdono 7, 20122 Milan, Italy

**Keywords:** arthroplasty, hip, hospital records, knee, length of stay, osteoarthritis, rehabilitation, retrospective studies

## Abstract

**Background**: Medical progress and sustainability pressures have made reducing hospital Length Of Stay (LOS) for total joint arthroplasty increasingly feasible and necessary. Monitoring rehabilitation duration and outcomes after surgical ward discharge needs equal attention. The aim of this retrospective, cohort study is to evaluate perioperative predictors of Inpatient Rehabilitation LOS (IRLOS), Discharge Destination (DD) (home versus residential care unit) and Need for Assistance at Discharge (NAD), in patients undergoing inpatient rehabilitation after total hip or knee arthroplasty in a high-volume, specialized research hospital. **Methods**: Electronic hospital datasets were employed to identify all adults with hip or knee osteoarthritis who received specialistic inpatient rehabilitation after total joint replacement between January and December 2019. Associations between demographic, clinical, surgical and functional variables and postoperative outcomes were examined using binary logistic regression for dichotomous outcomes (DD, NAD) and linear regression for continuous outcomes (IRLOS). **Results**: Based on a cohort of 1679 patients, we found various patient-related (age, working status, living alone, pre-existing comorbidities, osteoarthritic characteristics), surgical (duration of intervention, LOS, joint approach) and postoperative (hemoglobin levels, functional status) predictors. Overall, the regression models explained a modest but meaningful proportion of the variability in rehabilitation duration and post-discharge outcomes (R^2^ ranging from 0.12 to 0.34), resulting in marginal changes compared to a preliminary version of the same study on a smaller dataset. **Conclusions**: External validation on another cohort from the same hospital could be used to test the model’s predictivity at the local level, supporting the continuity of care between an orthopedic hospital hub and outpatient care and rehabilitation. Gains in predictive capacity may follow from including local factors like the operating surgeon and team. Although these factors could significantly improve the model performance at the local level, they would not be generalizable in different settings.

## 1. Introduction

Osteoarthritis of the hip and knee is among the most disabling musculoskeletal conditions worldwide, and its prevalence is expected to rise with population aging and increasing obesity rates [1]. Total joint replacement is indicated to relieve pain and disability in end-stage hip and knee osteoarthritis [2].

Medical progress and sustainability pressures have made reducing hospital Length Of Stay (LOS) for total joint replacement increasingly feasible and necessary, implementing accelerated protocols like fast-track [3], enhanced recovery after surgery [4], short stay [5] and same-day discharge [6] programs. These programs can reduce surgical waiting lists while maintaining safety [7] and achieving non-inferior or better outcomes [8,9,10,11].

Monitoring rehabilitation duration and outcomes after surgical ward discharge needs equal attention. Local healthcare organization features, socio-economic determinants of care access and compliance, and ultimately patients’ ability to cope with potentially fragmented healthcare pathways are key roles in postoperative rehabilitation effectiveness and continuity [12,13,14], even more so in case of older, possibly frail adults, affected by other chronic conditions, impaired mobility and/or cognitive decline [15,16,17]. The need to predict length of recovery and outcomes of total hip and knee procedures is important to take organizational and therapeutic decisions as early as possible, from planning appropriate intervention (e.g., pain and blood management, iron diet, patient mobilization), to efficiently managing acute and intermediate hospital beds, and activating continuity of care in support of safe patient discharge [18]. However, most of the published studies focus on small sets of variables collected from preoperative assessment or surgical ward stay only [18,19,20,21,22], possibly overlooking broader connections between preoperative patient characteristics, surgical intervention, postoperative conditions, patient mobilization and inpatient rehabilitation [23].

In a previous study that we have performed in a specialized research hospital in northern Italy, age, living with a family, occupational status, baseline Barthel Index of Autonomy in the Activities of Daily Living (ADL) and duration of surgery demonstrated a significant predictive value towards inpatient rehabilitation stay (IRLOS) and burden of care at discharge, in patients who underwent joint replacement for hip and knee osteoarthritis, based on linear and logistic regression models [24]. Although the proportion of explained variance (R^2^) was moderate, the identified predictors demonstrated good internal consistency and clinical plausibility.

In this follow-up study, we tested the hypothesis that additional perioperative variables, collected upon clinical advice and literature review, provided meaningful incremental explanatory value over the previously established predictors. In addition, discharge destination (DD) (home versus institutional setting) was included as a further outcome to broaden the evaluation of post-rehabilitation pathways.

## 2. Materials and Methods

### 2.1. Study Design

The manuscript is reported following the STROBE (STRengthening the reporting of OBservational studies in Epidemiology) checklist for cohort studies (Supplementary File S1). The study was conducted in accordance with the Declaration of Helsinki and was approved by the local Ethical Committee (See Institutional Review Board Statement).

The study is a secondary analysis of a larger set of clinical variables of patients who underwent inpatient rehabilitation after surgery at the IRCCS Galeazzi-Sant’Ambrogio Hospital in Milan, Lombardy, Italy [24].The additional variables collected were surgical access technique, pre-existing comorbidities, pre-existing pharmacological treatments and additional pharmacological treatment (as chronic use of a drug can reveal a pre-existing condition not reported by the patient in the medical history). The amount of care needed by patients at home or residential care units, previously called “Burden of care at discharge”, was named here as “Need of Assistance at Discharge (NAD)”, as we believe that this label describes such an outcome more clearly.

Electronic hospital datasets were employed to identify all adult patients with hip or knee osteoarthritis who underwent specialized inpatient rehabilitation after total joint arthroplasty between January and December 2019, at the same hospital. The diagnoses and procedures of interest were identified based on the International Classification of Disease, 9th Revision, Clinical Modification (ICD-9CM) codes 715.15, 715.16, 715.25, and 715.26 (diagnostic codes), 81.51 and 81.54 (procedure codes). Only participants with complete data were considered.

Once these patients were identified, five researchers (FP, LI, SB, MV, VT) retrieved data from surgical and rehabilitation clinical records. The data were collected between December 2023 and September 2025. The internal inpatient identifier allowed tracking the entire patient pathway from pre-operative surgical assessment to discharge (Table 1).

### 2.2. Variables

IRLOS was measured in days; DD was categorized as home or institutional setting; NAD was categorized as no need for ADL assistance or some degree of ADL assistance daily, based on the standardized information recorded by the Physical and Rehabilitation Medicine physician on the Hospital Discharge Form.

### 2.3. Statistical Analyses

All analyses were performed using SPSS 30 (IBM, Armonk, NY, USA). Continuous variables were summarized as mean ± standard deviation or median and interquartile range, as appropriate, while categorical variables were expressed as counts. The association between clinical, surgical, and functional variables with postoperative outcomes was assessed using binary logistic regression for dichotomous outcomes (NAD: no vs. yes; DD: home vs. institution) and linear regression for continuous outcomes (IRLOS). For all models, a backward stepwise variable selection approach was applied, with variables sequentially removed based on a threshold *p*-value > 0.10. Multicollinearity was assessed using Variance Inflation Factors (VIF), with values > 5 considered indicative of problematic collinearity. As VIF is not directly available for logistic regression in SPSS, we evaluated multicollinearity for logistic models using linear regression proxy models, including the same predictors as in the final logistic models. For linear regression models, VIFs were computed directly. Values > 5 were considered indicative of problematic collinearity.

In linear regression models, residuals were visually inspected for normality and homoscedasticity, showing no major violations of linear regression assumptions. In logistic regression models, model fit was assessed using Hosmer–Lemeshow tests.

All continuous variables were rescaled to a 0–1 range before inclusion in the regression models to enhance stability. Variables measured on standardized scales (e.g., Barthel Index of ADL) were divided by their maximum possible score, whereas other continuous variables were rescaled using clinically plausible upper bounds observed in the dataset. Statistical significance was set at *p* < 0.05.

For linear regression models, standardized regression coefficients (β) are reported to facilitate comparison of effect sizes across predictors, while results of logistic regression models are presented as odds ratios (Exp (B)). Statistical significance was set at *p* < 0.05. Model explanatory capacity was summarized using R^2^ for linear models and Nagelkerke R^2^ for logistic models.

As an internal validation step, nonparametric bootstrapping (1000 resamples, bias-corrected and accelerated confidence intervals) was applied to the final regression models re-estimated using an enter method, to descriptively assess coefficient variability after data-driven variable selection.

Given the substantial overlap between the present cohort and our previously published analyses, the primary aim of the modeling strategy was not to develop or optimize predictive models, but to assess whether the inclusion of additional perioperative variables provided incremental explanatory value beyond established predictors. In this context, changes in R^2^ (and Nagelkerke R^2^ for logistic models) were used as a pragmatic and transparent indicator of incremental value, allowing direct comparison with earlier models derived from the same population. This approach was chosen to quantify whether added model complexity resulted in meaningful gains in explained variance, rather than to maximize predictive performance.

## 3. Results

**Figure 1:**
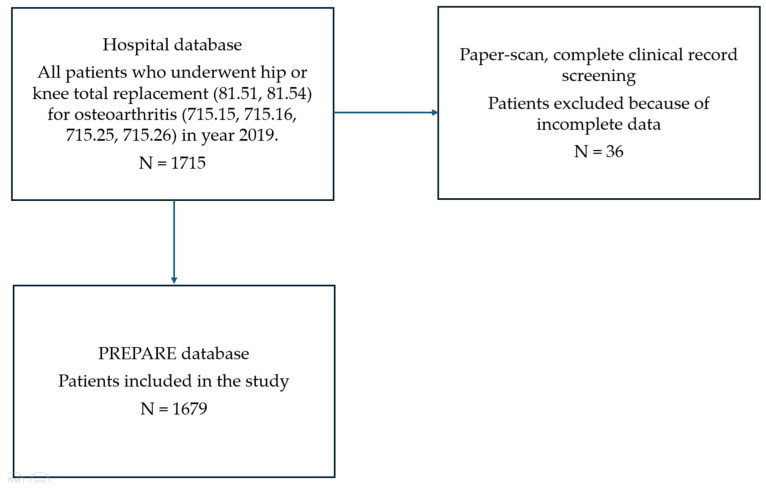
Patient’s selection flowchart.

Out of 1710 patients, we included 1679 patients after removing incomplete records (Figure 1).

Patients with incomplete records were excluded (Figure 1).

We found one more complete record in comparison to the previous study. Of these 1679 patients, 1070 patients (64%) had total knee arthroplasty and 609 patients (36%) had total hip arthroplasty. To improve clarity and impact, we reported only significant predictors narratively. Full predictors and analyses are available from Supplementary File S2.

Overall, the regression models explained a modest but meaningful proportion of the variability in rehabilitation duration and post-discharge outcomes (R^2^ ranging from 0.12 to 0.34).

### 3.1. Significant Predictors of Rehabilitation Outcomes After Total Knee Arthroplasty

IRLOS increased with patient age, longer surgery duration, longer surgical LOS, higher number of pre-existing comorbidities, and lower hemoglobin levels at rehabilitation admission. IRLOS decreased when a midvastus surgical approach was performed (Table 2).

After inpatient rehabilitation, patients were more likely to be discharged to an institutional setting when they lived alone, when a midvastus surgical approach was performed, with a longer rehabilitation LOS and lower functional independence at rehabilitation discharge, measured with the Barthel Index of ADL total score (Table 3).

After inpatient rehabilitation discharge, patients were more likely to need extended assistance in association with inactive working status, shorter surgical and rehabilitation LOS, longer surgery duration, lower hemoglobin levels at rehabilitation admission, midvastus surgical approach, and lower functional independence both at rehabilitation admission and discharge, measured with the Barthel Index of ADL total score (Table 4).

### 3.2. Significant Predictors of Rehabilitation Outcomes After Total Hip Arthroplasty

IRLOS increased with patient age, longer surgical time and number of pre-existing comorbidities. IRLOS decreased with higher functional independence at rehabilitation admission, measured with the Barthel Index of ADL total score, and posterolateral surgical approach (Table 5).

After an inpatient rehabilitation stay, patients were more likely to be discharged to an institutional setting in the presence of primary hip osteoarthritis, shorter surgical LOS, higher number of comorbidities, older age, and lower functional independence at hospital discharge (Table 6).

After inpatient rehabilitation discharge, patients were more likely to need extended assistance outside the hospital in association with shorter surgical LOS and lower functional independence at rehabilitation admission, measured with the Barthel Index of ADL total score (Table 7).

Bootstrap analyses showed broadly consistent coefficient estimates across resamples, although some predictors were characterized by wider confidence intervals.

## 4. Discussion

The regression models explained a modest but meaningful proportion of the variability in rehabilitation duration and outcomes (R^2^ ranging from 0.12 to 0.34), highlighting several predictive factors across patient characteristics, surgical details, and postoperative clinical measures. The inclusion of additional variables allowed us to refine the understanding of which factors most strongly influence both functional recovery and the need for support after discharge, while also confirming some findings previously reported in the literature.

When compared with previously published models based on the same cohort, the explanatory capacity of the models remained largely stable. Specifically, the R^2^ for inpatient rehabilitation LOS after total hip replacement remained unchanged (0.21 in both analyses), while a minimal increase was observed after total knee replacement (from 0.25 to 0.27). Similarly, the explained variance for post-discharge burden of care showed negligible differences for hip arthroplasty (from 0.13 to 0.12) and a modest increase for knee arthroplasty (from 0.20 to 0.26). Overall, the inclusion of additional perioperative variables resulted in only limited changes in R^2^, indicating marginal incremental explanatory value beyond established predictors.

With respect to functional variables, particularly the Barthel Index assessed at different time points along the care pathway, we acknowledge that a partial conceptual overlap with downstream outcomes such as discharge destination or need for assistance cannot be entirely excluded. In the context of inpatient rehabilitation, however, functional status measured at admission or discharge does not represent an independent determinant in a strict causal sense, but rather a clinically meaningful marker of the patient’s recovery process and functional reserve at a given stage. As such, these variables should be interpreted as indicators of recovery trajectories that inform discharge planning and care transitions, rather than as isolated predictors operating independently from the outcomes they help characterize.

### 4.1. Inpatient Rehabilitation After Total Knee Arthroplasty

Among patients undergoing total knee replacement, IRLOS increased with older age, longer surgical duration, longer surgical stay, a higher number of pre-existing comorbidities, and lower hemoglobin levels at rehabilitation admission. These findings align with previous literature showing slower recovery in older patients, those with more comorbidities, and individuals undergoing more complex or prolonged surgery [18,20]. Conversely, performing a midvastus surgical approach was associated with shorter IRLOS, likely reflecting the minimally invasive nature of the procedure. This result is consistent with prior studies, both smaller [20] and larger [21], reporting faster recovery, less pain, and higher Forgotten Joint Scores compared with medial or lateral parapatellar approaches.

Discharge to an institutional setting was more likely among patients living alone, those with longer rehabilitation LOS, lower functional independence at rehabilitation discharge (Barthel Index), and, unexpectedly, those who underwent a midvastus approach. Similarly, post-discharge need for ADL assistance was higher in patients with inactive working status, longer surgical duration, shorter surgical and rehabilitation LOS, lower hemoglobin at rehabilitation admission, midvastus approach, and lower functional independence at both rehabilitation admission and discharge. These results highlight that both baseline patient characteristics and perioperative factors contribute to post-discharge care needs.

The observed association between midvastus approach and higher likelihood of institutional discharge and post-discharge ADL assistance appears counterintuitive, given the previously reported faster recovery and non-inferior long-term outcomes at 45 days [21] or three years post-surgery [20]. This discrepancy may reflect unmeasured confounding factors not captured in our models, such as the operating surgeon or the assigned surgical ward [22].

Living alone was specifically associated with higher odds of institutional discharge after knee (but not hip) arthroplasty. In addition, longer rehabilitation LOS and lower functional independence at discharge further increased this risk. These findings are in line with prior evidence suggesting that functional improvements after hip arthroplasty are generally more substantial and stable than after knee arthroplasty [25], as measured by Clinician- and Patient-Reported Outcome Measures (CROMs and PROMs) and performance tests [2,26].

While age was not identified as a predictor, inactive working status increased the likelihood of requiring ADL assistance after knee, but not hip, arthroplasty. This may reflect the generally lower functional recovery observed after total knee replacement compared with total hip replacement, and patients motivated to return to work may achieve greater autonomy and tolerate residual knee symptoms better than those without occupational urgency. The influence of working status on post-operative recovery is supported by studies specifically investigating the predictive role of job intensity, status and satisfaction [27,28], although these factors were not investigated in combination with a broader set of perioperative data. Our findings confirm the importance of managing patient expectations when giving pre-arthroplasty information [28], even more in combination with other patient characteristics. Moreover, patients who were actively working before surgery are likely to have a higher preoperative function, which is consistent with the results of patients with a lower functional independence before surgery having a higher need for assistance at discharge. Pre-operative function is a predictor of functional outcomes both in hip [29] and knee arthroplasty [30] for osteoarthritis, with higher pre-operative function associated with higher post-operative function, although the variations may be more significant in patients with a lower baseline [29].

The association between lower hemoglobin levels at rehabilitation admission and more difficult functional recovery has given different results depending on what functional metric is adopted, e.g., Functional Independence Measure (FIM) or movement performance [31,32,33]. Our findings confirm an association between lower hemoglobin levels and slower functional recovery, expressed by a higher need for assistance with the ADL at discharge.

Finally, the inclusion of comorbidities and pre-existing pharmacological treatments resulted in minimal changes in R^2^ compared with the previous study, suggesting that these variables do not substantially enhance model explanatory power beyond core demographic, functional, and surgical predictors.

### 4.2. Inpatient Rehabilitation After Total Hip Arthroplasty

In patients undergoing total hip replacement, IRLOS increased with older age, longer surgical duration, and a higher number of pre-existing comorbidities, while higher functional independence at both rehabilitation admission and discharge (Barthel Index) was associated with shorter IRLOS. These findings are consistent with previous literature and expected clinical recovery patterns [34].

Discharge to an institutional setting was more likely in patients with longer surgical LOS, a higher number of comorbidities, and in those with primary, localized hip osteoarthritis (ICD-9CM 715.15) compared to secondary, localized osteoarthritis (ICD-9CM 715.25). This may be explained by younger age and more recent traumatic onset in secondary osteoarthritis [35], versus gradual, progressive functional deterioration in primary osteoarthritis, which can prolong recovery due to chronic pain, muscle weakness, and loss of function.

Regarding the surgical approach, a posterolateral approach was associated with shorter IRLOS compared to other approaches. Although this finding differs from some recent literature [36,37,38,39], early recovery differences may reflect unmeasured confounders, such as surgeon assignment or baseline severity of joint damage, rather than the approach itself. Considering the influence of pre-operative conditions on total hip replacement outcomes [40], patients with a higher degree of joint damage could be assigned to surgeons more prone to opt for a different hip access than the posterolateral approach, which means that the higher degree of joint damage could be responsible for worse outcomes, rather than the specific approach adopted or the surgeon.

Post-discharge ADL assistance needs were higher in patients with longer surgical LOS and lower functional independence at rehabilitation admission. Interestingly, longer surgical LOS predicted ADL assistance after hip replacement but did not prolong IRLOS, whereas for knee replacement, both longer surgical LOS and longer IRLOS were associated with higher post-discharge assistance needs. This likely reflects that prolonged surgical LOS after hip replacement may indicate complications or slower stabilization, increasing post-discharge assistance needs regardless of subsequent rehabilitation duration.

As with knee replacement, a higher number of comorbidities, but not pre-existing pharmacological treatment, contributed to improving the predictive models in comparison to the preliminary evaluation with the smaller dataset, confirming the importance of comorbidity burden in rehabilitation outcomes [24].

### 4.3. Limitations

A first limitation of this study is the modest explanatory capacity of the models, as reflected by relatively small R^2^ values. Although several predictors reached statistical significance, the proportion of variance explained remained limited, indicating that the models capture only part of the multifactorial processes underlying inpatient rehabilitation length of stay and post-discharge outcomes.

In addition, logistic regression models showed evidence of limited calibration, as indicated by significant Hosmer–Lemeshow tests. This result should be interpreted in light of the large sample sizes of the analyzed cohorts and the modest explanatory capacity of the models, as the Hosmer–Lemeshow test can be highly sensitive in such conditions. Importantly, these models were not intended for individual-level prediction, but to explore associations and incremental explanatory value within the care pathway. Moreover, although bootstrapping suggested no major coefficient instability, this approach does not account for uncertainty in the data-driven variable selection process inherent to backward stepwise procedures and should therefore be interpreted as descriptive rather than confirmatory.

In this study, we expanded the analytical focus from the surgical ward to the entire hospital stay, including inpatient rehabilitation duration and outcomes, with the aim of better informing clinicians, policy-makers and patients to plan and facilitate safer transitions to intermediate, outpatient, primary or home care. However, inpatient rehabilitation outcomes were treated primarily as endpoints or short-term indicators (e.g., DD and NAD), rather than as predictors of longer-term recovery trajectories. This represents an additional limitation of the present study.

An important implication of this finding is that inpatient rehabilitation data may be better conceptualized as intermediate markers within the recovery process rather than as isolated endpoints. Future studies could therefore explore their role as predictors of long-term outcomes, leveraging integrated health records that unify pre-admission data, hospital stays across different wards, and outpatient follow-up within a patient-centered clinical pathway. Such integration is likely essential to improve the explanatory and predictive performance of rehabilitation models and to support population stratification strategies relevant for clinical and policy decision-making.

While collecting PROMs would substantially enhance the assessment of patient-perceived changes, collecting Patient-Reported Experience Measures (PREMs) may be particularly informative in the context of care transitions, capturing aspects such as communication quality, patient involvement, and advanced planning for the post-hospital care, which are often most impacted by fragmentation and discontinuity of care [10,41,42,43].

Second, the operating surgeon and team could be confounding factors whose inclusion could potentially improve model performance. Including these factors could also explain some counterintuitive findings in comparison to findings in the literature. At the same time, having excluded the surgeon and the equipe should make the model more generalizable in different settings. Finally, the lack of external validation remains a limitation; validation on an independent cohort could help clarify the extent to which these contextual factors influence model performance.

The bootstrap results support a cautious interpretation of the observed associations, reinforcing the exploratory intent of the analyses rather than suggesting readiness for clinical prediction.

## 5. Conclusions

This study identified a list of patient-related (age, working status, living alone, pre-existing comorbidities, osteoarthritic characteristics), surgical (duration of intervention, LOS, joint approach), and postoperative (hemoglobin levels, functional status) predictors of IRLOS, DD, and NAD. These factors can support patient stratification strategies, the management of hospital beds, patients’ and caregivers’ expectations, and planned transitions to other settings. External validation on another cohort from the same hospital could be used to test the model’s predictivity at the local level, supporting the continuity of care between an orthopedic hospital hub and outpatient care and rehabilitation.

Although additional perioperative variables were included, the overall explanatory capacity of the models remained comparable to that of previously published analyses. The bootstrap results support a cautious interpretation of the observed associations, reinforcing the exploratory intent of the analyses rather than suggesting readiness for clinical prediction. Many statistically significant predictors have small effects and limited clinical utility. Significant gains in predictive capacity may follow from including local factors like the operating surgeon and team. Although these factors could significantly improve the model’s performance on a local level, they would not be generalizable in different settings.

## Figures and Tables

**Table 1:** Data sources linked per internal, anonymous patient identifier.

Administrative Database	Surgical Ward Records	Inpatient Rehabilitation Records
Patient identifier. Clinical records (surgical ward and inpatient rehabilitation) codes. Age at admission (number). Sex (male or female). Profession (multiple standardized options).	Main diagnosis (ICD-9CM). Main procedure (ICD-9CM). Body-Mass Index (BMI) before surgery. American Society of Anesthesiology (ASA) Score. Blood-borne infection (yes/no). Blood transfusion performed (yes/no). Day of Surgery (Monday-Saturday). Type of anesthesia (more options). **Surgical access technique** (more options). Duration of surgery (minutes). Surgical ward LOS (number of days).	Patient-reported pain at admission (NRS). Hemoglobin at admission (g/Dl). Barthel Index of ADL, total score at admission (0–100). Cognitive function at admission: memory domain (0–7), relations domain (0–7), problem-solving domain (0–7). Living arrangement (more standard options). **Pre-existing comorbidities** (more options). **Pre-existing pharmacological treatment** (yes, covering all reported pre-existing comorbidities, if pharmacological treatment is appropriate; no; partial; non-applicable, if no pre-existing comorbidities are reported). **Additional pharmacological treatment, based on reported pre-existing comorbidities** (yes/no). Barthel Index of ADL, total score at discharge (0–100). Cognitive function at discharge, three domains (0–7) each. Need for assistance (more standard options, in hours). Inpatient rehabilitation LOS (number of days).

The additional variables that were analyzed in the follow-up study are reported in bold. ADL: Activities of Daily Living. g/Dl: Grams per deciliter. ICD-9CM: International Classification of Diseases, 9th Revision—Clinical Modification. LOS: Length of Stay. NRS: Numeric Rating Scale.

**Table 2:** Inpatient rehabilitation length of stay, total knee arthroplasty.

	R^2^ = 0.27		
	Standardized β	*p*	VIF
Midvastus surgical approach	−0.20	**<0.001**	1.40
Age	0.18	**<0.001**	1.10
Surgical ward length of stay	0.06	**0.04**	1.34
Surgical time	0.28	**<0.001**	1.43
Living alone	0.05	0.08	1.06
Number of comorbidities	0.07	**0.01**	1.08
Hemoglobin	−0.09	**0.002**	1.16

Predictors of inpatient rehabilitation ward length of stay in people undergoing knee replacement surgery were assessed using a linear regression model. Significant *p* values (*p* < 0.05) are reported in bold. VIF: Variance Inflation Factor.

**Table 3:** Discharge destination; total knee arthroplasty.

	R^2^ = 0.12		
	Exp (B)	*p*	VIF
Living alone	2.4	**0.03**	1.01
Inpatient rehabilitation ward length of stay	1517.3	**<0.001**	1.16
Barthel Index (at rehabilitation discharge)	0.01	**0.01**	1.00
Midvastus surgical approach	2.45	**0.03**	1.16

Predictors of social outcome (home or institution discharge) in people undergoing knee replacement surgery assessed using a binary logistic regression model. Significant *p* values (*p* < 0.05) are reported in bold. VIF: Variance Inflation Factor. Hosmer-Lemeshow test: *p* = 0.39.

**Table 4:** Need for assistance at discharge, total knee arthroplasty.

	R^2^ = 0.26		
	Exp (B)	*p*	VIF
Working activity	0.69	**0.02**	1.04
Surgical ward length of stay	0.004	**<0.001**	1.40
Surgical time	10.99	**0.04**	1.38
Barthel Index (at rehabilitation admission)	0.06	**<0.001**	1.41
Hemoglobin	0.08	**0.03**	1.17
Rehabilitation ward length of stay	0.02	**<0.001**	1.32
Barthel Index (at rehabilitation discharge)	0.003	**0.04**	1.36
Parapatellar surgical approach	0.34	**<0.001**	1.32

Predictors of post-discharge care burden (no assistance or assistance) in people undergoing knee replacement surgery assessed using a binary logistic regression model. Significant *p* values (*p* < 0.05) are reported in bold. VIF: Variance Inflation Factor. Hosmer-Lemeshow test: *p* < 0.001.

**Table 5:** Inpatient rehabilitation length of stay, total hip arthroplasty.

	R^2^ = 0.21		
	Standardized β	*p*	VIF
Anterior surgical procedure	−0.16	0.06	5.38
Posterolateral surgical procedure	−0.17	**0.045**	5.45
Age	0.21	**<0.001**	1.79
Working activity	0.09	0.06	1.54
Surgical time	0.35	**<0.001**	1.11
Barthel Index (at rehabilitation admission)	−0.11	**0.004**	1.09
Number of comorbidities	0.11	**0.007**	1.06

Predictors of inpatient rehabilitation ward length of stay in people undergoing hip replacement surgery assessed using a linear regression model. Significant *p* values (*p* < 0.05) are reported in bold. VIF: Variance Inflation Factor. Moderately elevated VIF values observed for alternative surgical approach variables were expected, given their mutual exclusivity, and were therefore retained in the models.

**Table 6:** Discharge destination. Total hip arthroplasty.

	R^2^ = 0.34		
	Exp (B)	*p*	VIF
Primary osteoarthrosis	0.05	**<0.001**	1.05
Age	1219.7	**0.008**	1.20
Surgical ward length of stay	0.0	**0.007**	1.00
Biological risk	0.0	0.99	1.04
Number of comorbidities	41.8	0.06	1.18
Barthel Index (at rehabilitation discharge)	0.0	**<0.001**	1.06

Predictors of social outcome (home or institution discharge) in people undergoing hip replacement surgery assessed using a binary logistic regression model. Significant *p* values (*p* < 0.05) are reported in bold. VIF: Variance Inflation Factor. Hosmer-Lemeshow test: *p* = 0.83.

**Table 7:** Need for assistance at discharge. Total hip arthroplasty.

	R^2^ = 0.12		
	Exp (B)	*p*	VIF
Anterior surgical procedure	3.06	0.07	1.00
Surgical ward length of stay	0.01	**0.001**	1.01
Barthel Index (at rehabilitation admission)	0.01	**<0.001**	1.01

Predictors of post-discharge care burden (no assistance or assistance) in people undergoing hip replacement surgery assessed using a binary logistic regression model. Significant *p* values (*p* < 0.05) are reported in bold. VIF: Variance Inflation Factor. Hosmer-Lemeshow test: *p* = 0.005.

## Data Availability

The dataset is available at https://zenodo.org/records/18254857 (accessed on 6 February 2026).

## Supplementary Materials

The following supporting information can be downloaded at: https://www.mdpi.com/article/10.3390/jcm15062294/s1, File S1: STROBE Statement; File S2: Demographic Table.

## Abbreviations

The following abbreviations are used in this manuscript:

ADL	Activities of Daily Living
ASA	American Society of Anesthesiologists Score
BMI	Body-Mass Index
DD	Discharge Destination
ICD-9CM	International Classification of Disease, 9th Revision, Clinical Modification
IRLOS	Inpatient Rehabilitation Stay
LOS	Length of Stay
NAD	Need for Assistance at Discharge
NRS	Numeric Rating Scale
PREMs	Patient-Reported Experience Measures
PROMs	Patient-Reported Outcome Measures
STROBE	STRengthening the Reporting for Observational studies in Epidemiology
VIF	Variance Inflation Factors

## References

[1] World Health Organization (2023). Osteoarthritis. https://www.who.int/news-room/fact-sheets/detail/osteoarthritis.

[2] Judd D.L., Wolfe P., LeDoux C.V., Hogan C., Dayton M.R., Stevens-Lapsley J.E. (2019). Trajectories of functional performance and muscle strength recovery differ after total knee and total hip replacement: A performance-based, longitudinal study. Int. J. Rehabil. Res..

[3] Kehlet H. (2013). Fast-track hip and knee arthroplasty. Lancet.

[4] Riga M., Altsitzioglou P., Saranteas T., Mavrogenis A.F. (2023). Enhanced recovery after surgery (ERAS) protocols for total joint replacement surgery. SICOT J..

[5] Dawson-Bowling S.J., Jha S., Chettiar K.K., East D.J., Gould G.C., Apthorp H.D. (2014). A multidisciplinary enhanced recovery programme allows discharge within two days of total hip replacement; three- to five-year results of 100 patients. Hip Int..

[6] Jensen C.B., Troelsen A., Foss N.B., Nielsen C.S., Lindberg-Larsen M., Gromov K. (2023). 10-year evolution of day-case hip and knee arthroplasty: A Danish nationwide register study of 166,833 procedures from 2010 to 2020. Acta Orthop..

[7] Lan R.H., Samuel L.T., Grits D., Kamath A.F. (2021). Contemporary Outpatient Arthroplasty Is Safe Compared with Inpatient Surgery: A Propensity Score-Matched Analysis of 574,375 Procedures. J. Bone Jt. Surg. Am..

[8] Cheung A., Fu H., Cheung M.H., Chan W.K.V., Chan P.K., Yan C.H., Chiu K.Y. (2020). How well do elderly patients do after total knee arthroplasty in the era of fast-track surgery?. Arthroplasty.

[9] Berg U., W-Dahl A., Rolfson O., Nauclér E., Sundberg M., Nilsdotter A. (2020). Influence of fast-track programs on patient-reported outcomes in total hip and knee replacement (THR/TKR) at Swedish hospitals 2011-2015: An observational study including 51,169 THR and 8,393 TKR operations. Acta Orthop..

[10] Vanni F., Foglia E., Pennestrì F., Ferrario L., Banfi G. (2020). Introducing enhanced recovery after surgery in a high-volume orthopaedic hospital: A health technology assessment. BMC Health Serv. Res..

[11] Sarpong N.O., Boddapati V., Herndon C.L., Shah R.P., Cooper H.J., Geller J.A. (2019). Trends in Length of Stay and 30-Day Complications After Total Knee Arthroplasty: An Analysis From 2006 to 2016. J. Arthroplast..

[12] Lu Y., Khazi Z.M., Agarwalla A., Forsythe B., Taunton M.J. (2021). Development of a Machine Learning Algorithm to Predict Nonroutine Discharge Following Unicompartmental Knee Arthroplasty. J. Arthroplast..

[13] Seeber G.H., Wijnen A., Lazovic D., Bulstra S.K., Dietz G., van Lingen C.P., Stevens M. (2017). Effectiveness of rehabilitation after a total hip arthroplasty: A protocol for an observational study for the comparison of usual care in the Netherlands versus Germany. BMJ Open.

[14] Coudeyre E., Eschalier B., Descamps S., Claeys A., Boisgard S., Noirfalize C., Gerbaud L. (2014). Transcultural validation of the Risk Assessment and Predictor Tool (RAPT) to predict discharge outcomes after total hip replacement. Ann. Phys. Rehabil. Med..

[15] Lawless M.T., Marshall A., Mittinty M.M., Harvey G. (2020). What does integrated care mean from an older person’s perspective? A scoping review. BMJ Open.

[16] Brooks L., Stolee P., Elliott J., Heckman G. (2021). Transitional Care Experiences of Patients with Hip Fracture Across Different Health Care Settings. Int. J. Integr. Care.

[17] Theodorakis N., Kollia Z., Christodoulou M., Nella I., Spathara A., Athinaou S., Triantafylli G., Hitas C., Anagnostou D., Kreouzi M. (2025). Barriers to Implementing Effective Healthcare Practices for the Aging Population: Approaches to Identification and Management. Cureus.

[18] Pennestrì F., Banfi G. (2025). Predictive Factors of Inpatient Rehabilitation Stay After Elective Hip and Knee Replacement: A Scoping Review. Appl. Sci..

[19] Chen S., Qiang M., Li K., Wang X., Xie J., Wang W. (2024). Identifying patients at risk of prolonged hospital length of stay after total knee arthroplasty: A real-world study on the creation and validation of a cloud estimator. Biomol. Biomed..

[20] Lin W., Niu J., Dai Y., Yang G., Li M., Wang F. (2020). Mini-midvastus versus medial parapatellar approach in total knee arthroplasty: Difference in patient-reported outcomes measured with the Forgotten Joint Score. J. Orthop. Surg. Res..

[21] Blom A.W., Hunt L.P., Matharu G.S., Reed M., Whitehouse M.R. (2021). The effect of surgical approach in total knee replacement on outcomes. An analysis of 875,166 elective operations from the National Joint Registry for England, Wales, Northern Ireland and the Isle of Man. Knee.

[22] Song X., Xia C., Li Q., Yao C., Yao Y., Chen D., Jiang Q. (2020). Perioperative predictors of prolonged length of hospital stay following total knee arthroplasty: A retrospective study from a single center in China. BMC Musculoskelet. Disord..

[23] Shah A., Memon M., Kay J., Wood T.J., Tushinski D.M., Khanna V., McMaster Arthroplasty Collective (MAC) Group (2019). Preoperative Patient Factors Affecting Length of Stay following Total Knee Arthroplasty: A Systematic Review and Meta-Analysis. J. Arthroplast..

[24] Pennestrì F., Tosto V., Pelosi C., Grippa D., Negrini S., Kiekens C., Sarasso E., Banfi G., Cordani C., PREPARE Project Group (2024). Predictive Factors of Inpatient Rehabilitation Stay and Post-Discharge Burden of Care After Joint Replacement for Hip and Knee Osteoarthritis: A Retrospective Study on 1678 Patients. Appl. Sci..

[25] Naylor J.M., Harmer A.R., Heard R.C., Harris I.A. (2009). Patterns of recovery following knee and hip replacement in an Australian cohort. Aust. Health Rev..

[26] Blom A.W., Artz N., Beswick A.D., Burston A., Dieppe P., Elvers K.T., Gooberman-Hill R., Horwood J., Jepson P., Johnson E. (2016). Improving 466 Patients’ Experience and Outcome of Total Joint Replacement: The RESTORE 467 Programme.

[27] Sarfraz A., Antonioli S.S., Robin J.X., Rajahraman V., Schwarzkopf R., Arshi A., Rozell J.C. (2025). Does Physical Job Intensity Affect Return to Work and Satisfaction Rates Following Primary Total Hip Arthroplasty?. Arch. Orthop. Trauma. Surg..

[28] Kangas P., Soini S., Pamilo K., Kervinen V., Kinnunen M.L. (2025). Return to Work Following Hip or Knee Arthroplasty: A One-Year Prospective Cohort Study in Participants with Direct Referral from Hospital to Occupational Health Care Services. J. Occup. Rehabil..

[29] Hofstede S.N., Gademan M.G., Vliet Vlieland T.P., Nelissen R.G., Marang-van de Mheen P.J. (2016). Preoperative predictors for outcomes after total hip replacement in patients with osteoarthritis: A systematic review. BMC Musculoskelet. Disord..

[30] Jones C.A., Voaklander D.C., Suarez-Alma M.E. (2003). Determinants of function after total knee arthroplasty. Phys. Ther..

[31] Diamond P.T., Conaway M.R., Mody S.H., Bhirangi K. (2006). Influence of hemoglobin levels on inpatient rehabilitation outcomes after total knee arthroplasty. J. Arthroplast..

[32] Cavenaghi F., Cerri C., Panella L. (2009). Association of hemoglobin levels, acute hemoglobin decrease and age with Rehabilitation outcomes after total hip and knee replacement. Eur. J. Phys. Rehabil. Med..

[33] Wang X., Rintala D.H., Garber S.L., Henson H.K. (2005). Association of hemoglobin levels, acute hemoglobin decrease, age, and co-morbidities with rehabilitation outcomes after total knee replacement. Am. J. Phys. Med. Rehabil..

[34] Cleveland Clinic OME Arthroplasty Group (2021). Understanding the Main Predictors of Length of Stay After Total Hip Arthroplasty: Patient-Related or Procedure-Related Risk Factors?. J. Arthroplast..

[35] Ackerman I.N., Kemp J.L., Crossley K.M., Culvenor A.G., Hinman R.S. (2017). Hip and Knee Osteoarthritis Affects Younger People, Too. J. Orthop. Sports Phys. Ther..

[36] Christensen J.C., Judd D.L., Forster J.E., O’Malley S., Hinrichs-Kinney L., Hogan C.A., Dayton M.R., Christiansen C.L., Stevens-Lapsley J.E. (2025). Comparing Direct Anterior Approach Versus Posterolateral Approach in Total Hip Arthroplasty on Physical Function Recovery: A Prospective Cohort Study. J. Orthop. Res..

[37] Amouzadeh Omrani F., Afzal S., Baroutkoub M., Salimi S., Barati H., Azadnajafabad S., Kokly S., Omidian M.M. (2025). A Comparative Analysis of Functional Outcomes between Lateral and Posterolateral Approaches in Total Hip Arthroplasty. Adv. Biomed. Res..

[38] Stock L.A., Johnson A.H., Brennan J.C., MacDonald J., Turcotte J.J., King P.J. (2023). The Impact of Total Hip Arthroplasty Surgical Approach on Short-Term Postoperative and Patient-Reported Outcomes. Cureus.

[39] Wang Z., Bao H.W., Hou J.Z., Ju B., Wu C.H., Zhou Y.J., Gu X.M., Wang H.H. (2022). The Direct Anterior Approach versus the Posterolateral Approach on the Outcome of Total Hip Arthroplasty: A Retrospective Clinical Study. Orthop. Surg..

[40] Günther K.P., Deckert S., Lützner C., Lange T., Schmitt J., Postler A. (2021). Total Hip Replacement for Osteoarthritis-Evidence-Based and Patient-Oriented Indications. Dtsch. Arztebl. Int..

[41] Cordani C., Perillo S., Corbetta D., Sarasso E., Agosta F., Filippi M., Mazzali A.G., Pennestrì F. (2024). Developing Physiotherapy in Primary Health Care: A First Snapshot from the Italian Metropolitan City of Milan. Healthcare.

[42] Pennestrì F., Banfi G. (2022). The Experience of Patients in Chronic Care Management: Applications in Health Technology Assessment (HTA) and Value for Public Health. Int. J. Environ. Res. Public Health.

[43] Pennestrì F., Lega F., Banfi G. (2023). From volume to value: Improving peri-operative elective pathways through a roadmap from fast-track orthopedic surgery. Health Serv. Manag. Res..

