# Supplementary material for: Prediction of Inpatient Rehabilitation Length, Discharge Destination and Home-Care Needs After Total Hip and Knee Arthroplasty for Osteoarthritis: A Follow-Up Study on 1.679 Patients
Source: J Clin Med. 2026 Mar 17;15(6):2294. doi: 10.3390/jcm15062294 (PMC13026979; doi:10.3390/jcm15062294)
Supplement: Supplementary file 1 [file jcm-15-02294-s001.zip › jcm-4126832-supplementary.pdf]

Supplementary File S1

STROBE Statement—checklist of items that should be included in reports of observational studies

	Item No	Recommendation	Page No
**Title and abstract**	1	(*a*) Indicate the study's design with a commonly used term in the title or the abstract	1
		(*b*) Provide in the abstract an informative and balanced summary of what was done and what was found	1-2
**Introduction**			
Background/rationale	2	Explain the scientific background and rationale for the investigation being reported	2-3
Objectives	3	State specific objectives, including any prespecified hypotheses	2-3
**Methods**			
Study design	4	Present key elements of study design early in the paper	3
Setting	5	Describe the setting, locations, and relevant dates, including periods of recruitment, exposure, follow-up, and data collection	3
Participants	6	(*a*) *Cohort study*—Give the eligibility criteria, and the sources and methods of selection of participants. Describe methods of follow-up	3
		(*b*) *Cohort study*—For matched studies, give matching criteria and number of exposed and unexposed	N/A
Variables	7	Clearly define all outcomes, exposures, predictors, potential confounders, and effect modifiers. Give diagnostic criteria, if applicable	Table 1
Data sources/ measurement	8*	For each variable of interest, give sources of data and details of methods of assessment (measurement). Describe comparability of assessment methods if there is more than one group	4-5
Bias	9	Describe any efforts to address potential sources of bias	N/A
Study size	10	Explain how the study size was arrived at	5
Quantitative variables	11	Explain how quantitative variables were handled in the analyses. If applicable, describe which groupings were chosen and why	5
Statistical methods	12	(*a*) Describe all statistical methods, including those used to control for confounding	4-5
		(*b*) Describe any methods used to examine subgroups and interactions	N/A
		(*c*) Explain how missing data were addressed	N/A
		(*d*) *Cohort study*—If applicable, explain how loss to follow-up was addressed	N/A
		(*e*) Describe any sensitivity analyses	N/A
**Results**			
Participants	13*	(a) Report numbers of individuals at each stage of study—eg numbers potentially eligible, examined for eligibility, confirmed eligible, included in the study, completing follow-up, and analysed	5
		(b) Give reasons for non-participation at each stage	N/A
		(c) Consider use of a flow diagram	N/A

Descriptive data	14*	(a) Give characteristics of study participants (eg demographic, clinical, social) and information on exposures and potential confounders	Table 1
		(b) Indicate number of participants with missing data for each variable of interest	N/A
		(c) *Cohort study*—Summarise follow-up time (eg, average and total amount)	5
Outcome data	15*	*Cohort study*—Report numbers of outcome events or summary measures over time	N/A
		Case-control study—Report numbers in each exposure category, or summary measures of exposure	N/A
		Cross-sectional study—Report numbers of outcome events or summary measures	
Main results	16	(*a*) Give unadjusted estimates and, if applicable, confounder-adjusted estimates and their precision (eg, 95% confidence interval). Make clear which confounders were adjusted for and why they were included	Tables 2-7
		(*b*) Report category boundaries when continuous variables were categorized	N/A
		(*c*) If relevant, consider translating estimates of relative risk into absolute risk for a meaningful time period	N/A
Other analyses	17	Report other analyses done—eg analyses of subgroups and interactions, and sensitivity analyses	N/A
**Discussion**			
Key results	18	Summarise key results with reference to study objectives	5-8
Limitations	19	Discuss limitations of the study, taking into account sources of potential bias or imprecision. Discuss both direction and magnitude of any potential bias	8
Interpretation	20	Give a cautious overall interpretation of results considering objectives, limitations, multiplicity of analyses, results from similar studies, and other relevant evidence	8-9
Generalisability	21	Discuss the generalisability (external validity) of the study results	8
**Other information**			
Funding	22	Give the source of funding and the role of the funders for the present study and, if applicable, for the original study on which the present article is based	2

Supplementary File S2. Demographic Table

**Demographic Table**				
	**Hip (N=609)**		**Knee (N=1070)**	
	Mean	SD	Mean	SD
Age	68.3	11.5	69.8	9.5
BMI	27.1	4.9	28.9	5.0
Length of stay, surgery	5.0	1.5	4.7	1.3
ASA*	2	2-2	2	2-2
Surgical time	59.4	32.4	65.4	30.5
Barthel Index surgery	69.7	12.3	70.7	11.8
Pain - VNS	2.1	1.2	2.2	1.2
Number comorbidities*	1	1-2	1	1-2
Hemoglobin	10.1	1.3	10.7	1.5
Days to verticalization	1.1	0.7	1.1	0.7
Length of stay, rehabilitation	10.9	3.9	10.0	3.6
Barthel Index rehabilitation	94.8	7.3	96.2	5.7
Primary/secondary osteoarthrosis	580	29	1055	15
Surgical procedure (anterior/postero-lateral)	51	558	651	419
Gender (female/male)	343	266	709	361
Occupational status (unemployed/employed)	406	203	725	345
Anesthesia (general/others)	42	567	28	1042
Biological risk (yes/no)	3	606	22	1048
Blood transfusion (yes/no)	106	503	121	949
Live with (family/other)	489	120	889	181
Previous prosthesis (yes/no)	129	480	238	832
Cardiovascular comorbidities (yes/no)	377	232	750	320
Respiratory comorbidities (yes/no)	42	567	99	971
Neurological-Psychiatric comorbidities (yes/no)	49	560	74	996
Hematological comorbidities (yes/no)	12	597	25	1045
Metabolic comorbidities (yes/no)	80	529	155	915
Rheumatological-musculoskeletal comorbidities (yes/no)	38	571	72	998
Oncological comorbidities (yes/no)	10	599	11	1059
Renal comorbidities (yes/no)	13	596	13	1057
Sensory comorbidities (yes/no)	13	596	24	1046
Infective comorbidities (yes/no)	7	602	23	1047
Gastrointestinal comorbidities (yes/no)	3	606	9	1061
No comorbidities (yes/no)	147	462	202	868
Pharmacological therapies (yes/no)	431	178	813	257
Extra pharma (yes/no)	149	460	267	803

Discharge place (home/institution)	582	27	1038	32
Needs of assistance (yes/no)	495	114	793	277

(*) Data are reported as median and interquartile range
